# A longitudinal analysis of humoral, T cellular response and influencing factors in a cohort of healthcare workers: Implications for personalized SARS-CoV-2 vaccination strategies

**DOI:** 10.3389/fimmu.2023.1130802

**Published:** 2023-03-14

**Authors:** Eleonora Sabetta, Maddalena Noviello, Clara Sciorati, Marco Viganò, Rebecca De Lorenzo, Valeria Beretta, Veronica Valtolina, Chiara Di Resta, Giuseppe Banfi, Davide Ferrari, Massimo Locatelli, Fabio Ciceri, Chiara Bonini, Patrizia Rovere-Querini, Rossella Tomaiuolo

**Affiliations:** ^1^ Vita-Salute San Raffaele University, Milan, Italy; ^2^ Experimental Hematology Unit, Division of Immunology, Transplantation, and Infectious Diseases, IRCCS San Raffaele Scientific Institute, Milan, Italy; ^3^ Cell Therapy Immunomonitoring Laboratory (MITiCi), Division of Immunology, Transplantation, and Infectious Diseases, IRCCS San Raffaele Scientific Institute, Milan, Italy; ^4^ Innate Immunity and Tissue Remodeling Unit, Division of Immunology, Transplantation, and Infectious Diseases, IRCCS San Raffaele Scientific Institute, Milan, Italy; ^5^ Scientific Direction, IRCCS Orthopedic Institute Galeazzi, Milan, Italy; ^6^ SCVSA Department, University of Parma, Parma, Italy; ^7^ Laboratory Medicine Service, IRCCS San Raffaele Scientific Institute, Milan, Italy; ^8^ Hematology and Bone Marrow Transplant Unit, IRCCS San Raffaele Scientific Institute, Milan, Italy

**Keywords:** COVID-19, T-cell response, SARS-CoV-2 vaccination, serological tests and risk factors, influencing factors (variables)

## Abstract

**Introduction:**

SARS-CoV-2 mRNA vaccinations elicit both virus-specific humoral and T-cell responses, but a complex interplay of different influencing factors, such as natural immunity, gender, and age, guarantees host protection. The present study aims to assess the immune dynamics of humoral, T-cell response, and influencing factors to stratify individual immunization status up to 10 months after Comirnaty-vaccine administration.

**Methods:**

To this aim, we longitudinally evaluated the magnitude and kinetics of both humoral and T-cell responses by serological tests and enzyme-linked immunospot assay at 5 time points. Furthermore, we compared the course over time of the two branches of adaptive immunity to establish an eventual correlation between adaptive responses. Lastly, we evaluated putative influencing factors collected by an anonymized survey administered to all participants through multiparametric analysis. Among 984 healthcare workers evaluated for humoral immunity, 107 individuals were further analyzed to describe SARS-CoV-2-specific T-cell responses. Participants were divided into 4 age groups: <40 and ≥40 years for men, <48 and ≥48 years for women. Furthermore, results were segregated according to SARS-CoV-2-specific serostatus at baseline.

**Results:**

The disaggregated evaluation of humoral responses highlighted antibody levels decreased in older subjects. The humoral responses were higher in females than in males (p=0.002) and previously virus-exposed subjects compared to naïve subjects (p<0.001). The vaccination induced a robust SARS-CoV-2 specific T-cell response at early time points in seronegative subjects compared to baseline levels (p<0.0001). However, a contraction was observed 6 months after vaccination in this group (p<0.01). On the other hand, the pre-existing specific T-cell response detected in natural seropositive individuals was longer-lasting than the response of the seronegative subjects, decreasing only 10 months after vaccination. Our data suggest that T-cell reactiveness is poorly impacted by sex and age. Of note, SARS-CoV-2-specific T-cell response was not correlated to the humoral response at any time point.

**Discussion:**

These findings suggest prospects for rescheduling vaccination strategies by considering individual immunization status, personal characteristics, and the appropriate laboratory tests to portray immunity against SARS-CoV-2 accurately. Deepening our knowledge about T and B cell dynamics might optimize the decision-making process in vaccination campaigns, tailoring it to each specific immune response.

## 1 Introduction

Since the World Health Organization (WHO) declared a pandemic status for Coronavirus Disease 2019 (COVID-19) caused by Severe Acute Respiratory Syndrome Coronavirus 2 (SARS-CoV-2), total vaccine administered doses have been over 13 billion with almost 5.6 billion persons vaccinated with at least one dose (1). Despite the considerable efforts to bring the COVID-19 pandemic under control, vaccine distribution is still heavily skewed (2), and assessing the immunization status of recipients remains critical in streamlining worldwide vaccination strategies.

The adaptive immune system, with its two fundamental components, B and T cells, is involved in controlling SARS-CoV-2 infection (3), in viral clearance, and protection from reinfection following vaccination (4). The synthesis of antibodies by B cells starts early after SARS-CoV-2 infection (5), with activated naïve B cells cooperating with T cells in the germinal center (GC) to produce highly specific antibodies. GC-derived memory B cells and bone marrow-resident plasma cells provide long-lasting protection against reinfection, while short-lived peripheral plasma cells produce most antibodies during the acute infection. Consequently, the antibody titer declines with time, but memory B cells, upon re-exposure, rapidly expand and differentiate into antibody-secreting plasma cells (4). Furthermore, greater potency and breadth are reported in the case of infection-induced antibodies than vaccination-elicited ones, displaying differential evolution over time due to numerous factors, including the route of antigen delivery, the nature of the antigen, and antigen persistence. These aspects could influence B cell evolution and selection through differential T cell recruitment (6). To date, serological tests detecting the presence of SARS-CoV-2 antibodies have played a central role in epidemiological assessments and estimating global therapeutic needs (7). However, numerous limitations narrow their applicability in testing scenarios as they cannot determine whether an individual is currently infectious or protected against re-infection (8, 9). Moreover, serology testing is impaired by high intra/inter-laboratory variability (10) and lack of a neutralization threshold *in vitro* as a reliable correlate of protection (8).

Likewise, during SARS-CoV-2 infection, the T cellular response develops within the first weeks (4) and produces virus-specific CD8+ and CD4+ T cells, including CD8+ memory T cells (11). Specific memory T cells seem to play a crucial role in long-term immune protection against COVID-19 (12, 13), and mild disease is associated with more efficient T follicular helper responses in the GC, enhancing antibody production (14, 15). mRNA vaccinations are designed to induce a Th1-polarized cell response (14), and the evaluation of interferon-γ (IFN-γ) secretion is widely used as a diagnostic marker of effective T-cell immunity (e.g., with concern to Tuberculosis) (16, 17). The increasing automation and standardization of SARS-CoV-2-specific T-cell assays have expanded their diagnostic potential and applicability in real-world scenarios. Although maintaining time-consuming sample setups (e.g., The Interferon Gamma Releasing Assay test – IGRA test) (13) or requiring the use of viable cells (e.g., for the IFN-γ enzyme-linked immunospot assay - ELISpot assay), their role in routine laboratory practice is consolidating.

Since the immune response to vaccination and infection depends on individual characteristics, the complex interrelation among numerous factors (such as gender, age, and pre-existing immunity) (18, 19) may be crucial when planning a targeted vaccination strategy. Indeed, it was reported that females tend to have a more vigorous serological response to vaccines than males (18). At the menopause transition, the decrease of estradiol potentially intensifies immunosenescence, and aging women lose their immunological advantage displaying increased susceptibility and mortality to specific infections (20). Moreover, the adaptive immune response shows recognizable changes over time with aging: T-cell senescence and dysfunction (e.g., diminished acquired immune capacity, increasing pro-inflammatory traits, and a higher risk for autoimmunity) in the elderly may underlie the high susceptibility to develop severe infection (21) and may result in a reduction of antibody production and protective immunity following immunizations (22). Finally, a previous encounter with the virus determines a higher reactogenicity after the first dose, both for humoral (18) and T-cell responses (23).

The present study aims to assess the kinetics of humoral and T-cellular responses, analyze influencing factors potentially impacting the individual-specific immune responses, and suggest possible implications for personalized SARS-CoV-2 vaccination strategies. To this aim, we analyzed the magnitude and persistence of humoral and T-cell responses employing both serological tests and ELISpot assay at several time points up to 10 months after vaccination. Moreover, we evaluated the role of putative influencing factors on the individual-specific immune response by multiparametric analysis of data collected within an anonymized survey.

## 2 Materials and methods

### 2.1 Study design

In this longitudinal observational study, the kinetics of B- and T-cell responses were evaluated by serological tests and IFN-γ ELISpot in the blood of healthcare workers (HCWs), including physicians, nurses, laboratory staff, researchers, administrative personnel, and collaborators. All HCWs received the first and the second dose of the BNT162b2 mRNA COVID-19 Vaccine from January to June 2021 at IRCCS Ospedale San Raffaele (OSR). Peripheral blood samples were collected at five different time points: a) on day 0, a few minutes before the first administration of the vaccine (T0); b) 21 days after the first dose (a few minutes before administration of the second dose) to evaluate the response to the first dose (T1); c) 21 days after the second dose, to evaluate the immune response upon completion of the vaccination course (T2); d) 6 months after the first dose (T3); e) before the booster dose (10 months after the first dose; T4).

Demographic and clinical data were collected by an anonymized survey, and a multiparametric analysis evaluated putative influencing factors.

### 2.2 Specific humoral response kinetics

The humoral response kinetics was evaluated by serological test results collected from January to December 2021 within the COVIDIAGNOSTIX project (CE:199/INT/2020), approved by OSR Ethical Committee. All participants (n=984) signed specific written informed consent. Of these, 15 were excluded from humoral response analyses due to evidence of active SARS-CoV-2 infection between the first and second dose of the vaccine (n=969). Antibody titers were tested by the Elecsys Anti-SARS-CoV-2 assay (Roche, Basel, Switzerland) specific for the viral SARS-CoV-2 Nucleocapsid protein (N) at T0 and by the Elecsys SARS-CoV-2-S (Roche, Basel, Switzerland) against the RBD of the viral Spike (S) protein at T1, T2, T3, and T4. The Roche Elecsys Anti-SARS-CoV-2 is an electrochemiluminescence immunoassay (ECLIA) targeted on total immunoglobulins (IgTot: IgA, IgG, and IgM) against the N-protein. The result is given as a cut-off index (COI) and qualitative results: for COI 1.0, the sample is reactive and positive (manufacture datasheet: 09289267501V0.6). The manufacturer indicated a specificity of 99.80% and a sensitivity of 99.50% 14 days post-PCR confirmation. The Roche anti-SARS-CoV-2-S is an ECLIA detecting total immunoglobulins (IgTot: IgA, IgG, and IgM) against the RBD of the viral S-protein. The quantification range is between 0.4 and 250.0 U/mL, which is further extended to 2500.0 U/mL by a 1:10 dilution of the sample automatically performed by the instrument. Specificity and sensitivity (≥ 14 days after diagnosis) are 99.98% and 98.8%, respectively, when the manufacturer’s suggested COI for positivity 0.8 U/mL is used.

### 2.3 Specific T-cell response kinetics

Amongst all participants, 107 healthcare workers aged 51 (IQR: 37-62) were evaluated by the IFN-γ ELISpot to quantify SARS-CoV-2 specific T-cell responses (named ELISpot population in the manuscript). The ELISpot population signed an informed consent for the storage and usage of PBMC in the Institutional Biobank within the studies BIOVAC (CE:17/INT/2022) and BIOVAC-Immunity (CE: 64/INT/2022) approved by the OSR Ethical Committee. Frequencies of IFN-γ-producing SARS-CoV-2-specific T cells were evaluated using cryopreserved PBMC harvested at T0, T2, T3, and T4. Age-matched PBMC samples collected before the pandemic were also tested as unexposed controls (n=17). PBMC were thawed and kept in culture for two hours in IMDM (Lonza) supplemented with 10% Human Serum, Glutamine (1%), and Penicillin/Streptomycin (1%) in the presence of low doses of recombinant human IL-2 (rhIL-2, 20 UI/ml, Novartis). Cells were then washed to eliminate the rhIL-2. The Mabtech ELISpot kit was used, featuring pre-coated plates and one-step detection to increase reproducibility, according to the manufacturer’s instructions. 400’000 PBMC were seeded per well and stimulated for 16-20 hours with two libraries of overlapping peptides spanning the S and the N proteins: PepTivator SARS-CoV-2 S and N, respectively (1 μg of each peptide per ml; Miltenyi). Anti-CD3 monoclonal antibodies included in the Mabtech ELISpot kit were used as the positive control and irrelevant peptides as the negative control (pool of peptides covering the sequence of ovalbumin, 1 μg of each peptide per ml; Miltenyi). The anti-CD28 monoclonal antibody (1 μg/ml; BD) was added in each condition to provide costimulatory signals and increase T-cell stimulation. Spot-forming cells (SFC) were quantified by the ImmunoCapture 7.0 software (TLC ELISpot Reader). Unstimulated T cells and T cells stimulated with irrelevant peptides were subtracted. Results were expressed as specific SFC/400’000 PBMC.

### 2.4 Survey

A survey about demographics, anthropometrics, medical history, and adverse effects following the first and second doses of the vaccine was provided to all participants six months after vaccine administration. Medical history was focused on pre-existing comorbidities categorized into (a) neoplastic disease, either current or under treatment in previous years; (b) allergic pathologies; (c) diabetes mellitus (type 1 or type 2); (d) blood diseases (coagulopathies, anemia); (e) history of immunosuppression/organ transplant; (f) cardiovascular diseases; (g) neurological diseases; (h) autoimmune diseases; (i) infectious diseases (liver diseases, other); (l) smoking history; (m) chronic pharmacological therapy; (n) other diseases. The survey also gathers information on medical events related to COVID-19, e.g., whether the viral infection was contracted less or more than 6 months before vaccination. Moreover, the survey included questions on which symptoms were associated with SARS-CoV-2 infection: fever ≥37.5 °C, chills, cough, sore throat, rhinorrhea, otalgia, dyspnea, chest pain, anosmia, ageusia, myalgia, arthralgia, fatigue/asthenia, nausea/vomiting, diarrhea or abdominal pain, conjunctivitis, disturbance or loss of consciousness, epilepsy, skin rash or ulcers, bleeding. To assess the side effects of the SARS-CoV-2 vaccine, the survey incorporated questions about the incidence of (a) localized reactions (pain, swelling, redness, swelling at the injection site); (b) systemic reactions (fever, tiredness/malaise, chills, headache, vomiting/nausea, diarrhea, body aches, swollen lymph nodes, dizziness/confusion); (c) allergic reactions (widespread itching, rash other than the injection site, asthma, throat tightness, anaphylaxis); and (d) other reactions (sleep quality alteration, memory loss, anxiety, psychological stress, feeling of gratitude/relief/joy, attention deficit, palpitations, chest pain, appetite loss, increased thirst, heat/cold intolerance, menstrual cycle alterations, difficulty to perform daily life activities). Lastly, it was asked whether the HCWs were frontline workers during the first wave of the pandemic.

### 2.5 Outputs

Anti-N-SARS-CoV-2 antibody titers at T0 and anti-RBD-SARS-CoV-2 antibody titers at T1, T2, T3, T4 and IFN-γ quantification at T0, T2, T3, and T4 were used as primary outputs. Moreover, we evaluated the impact of putative influencing factors on humoral and T-cell response. The occurrence of at least one systemic reaction (i.e., fever, tiredness/malaise, chills, myalgias, arthralgias) following the first or the second dose of vaccine served as secondary outputs.

### 2.6 Variables for statistical analysis

The survey collected the following variables: age, sex, ethnicity, smoking history, BMI, professional category (frontline *vs.* non-frontline workers), pre-existing comorbidities, and self-reported adverse events following the first and the second dose of vaccine. With antibody testing at baseline (T0), subjects tested with anti-N-specific antibody titer >1 were considered naturally seropositive (having a history of SARS-CoV-2 infection before vaccination).

### 2.7 Statistical analysis

Descriptive statistics were performed for all variables. Continuous variables were expressed as median (IQR), while categorical variables as absolute counts (proportions [%]). Chi-squared and Mann-Whitney U tests were employed to assess the statistical significance of differences in categorical and continuous variables, respectively, between groups. For the analysis, the study population was divided into the following age groups: <48 and ≥48 years for women (based on the gradual decrease of estrogen with menopause) and <40 and ≥40 years for men (based on the gradual decrease of testosterone with aging in men). Kruskall-Wallis test with Dunn’s *post hoc* test and two-way ANOVA models for repeated measures were used to assess differences among different groups and/or follow-ups. Antibody titers and T-cell responses had a quasi-beta distribution after proper linear transformation to set the range between 0 and 1. Multiple beta regression models were subsequently used to evaluate the influence of specific independent variables on antibody titers (at T1, T2, T3, and T4) and T-cell responses at T2, T3, and T4, using the betareg package for R (24).

Multiple logistic regression models were built to identify variables predicting systemic reactions. One final logistic model was selected for each outcome based on the Akaike information criterion using the *multi*-package in R (25). All statistical tests were performed using the R statistical package v.4.1.0 (R Core Team, Vienna, Austria) or GraphPad Prism v.9.4.1 (GraphPad Software, San Diego, CA, USA). All tests were two-sided, with a significance level of p <0.05.

## 3 Results

### 3.1 Study population

All participants (n=984) filled in the survey concerning demographics, anthropometrics, medical history, and adverse reactions following the first and second dose of the vaccine. Out of these 984 subjects, 15 were excluded from the humoral response analyses due to evidence of active SARS-CoV-2 infection between the first and the second dose of the vaccine. The general characteristics of the 969 participants evaluated for humoral immunity are reported in **Supplementary Table 1**. The median age was 51 (interquartile range IQR: 41-58), and most subjects were female (65.4%). Seventy-seven (7.9%) subjects had a history of SARS-CoV-2 infection resolved before the first dose of the vaccine. The most common comorbidities were cardiovascular diseases (CVD, 20.1%) and autoimmune diseases (7.9%), mainly among females.

### 3.2 SARS-COV-2 Humoral response kinetics

**Figure 1:**
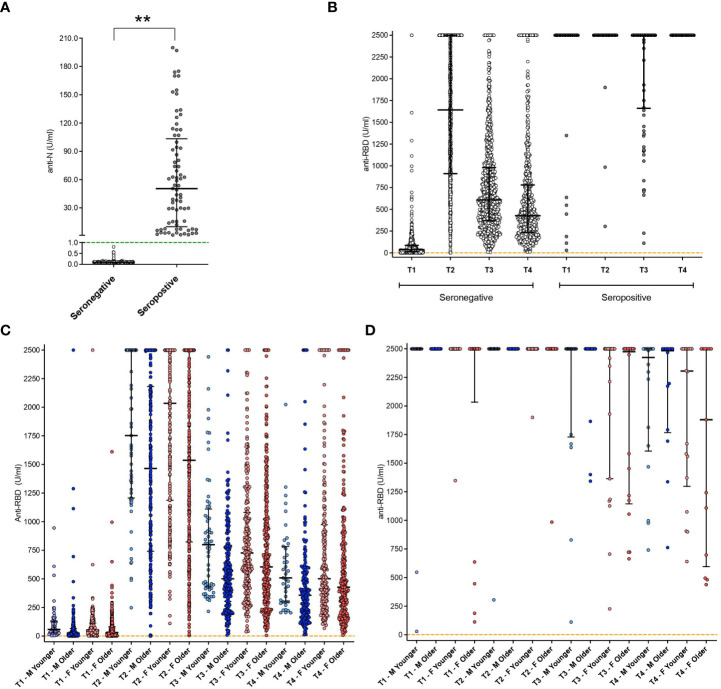
Antibody titers at different time points in the whole cohort. **(A)** Anti-Nucleocapsid (N) protein antibody titer results at baseline in both seropositive and seronegative subjects. Green dashed line represents the threshold for reactogenicity (1 U/mL); **: p<0.01. **(B)** anti-RBD antibodies aggregated results in both seropositive and seronegative subjects. Orange dashed line represents the threshold for reactogenicity (0.8 U/mL). **(C)** anti-RBD antibodies disaggregated data by gender and age groups (seronegative subjects). **(D)** anti-RBD antibodies disaggregated data by gender and age groups (seropositive subjects). Disaggregated evaluation allowed highlighting differences between males (M, in blue) and females (F, in red) and among different age groups (light blue: Males <40 y.o.; pink: Females < 48 y.o.; dark blue, Males ≥ 40 y.o.; red, females ≥ 48 y.o.). Individual dots represent each replicate. Median with interquartile range are shown.

Subjects showing anti-N-specific antibody titer >1 U/ml at baseline (T0) were considered naturally seropositive (having a history of SARS-CoV-2 infection before vaccination; **Figure 1A**).

Levels of anti-Receptor Binding Domain (RBD) SARS-CoV-2 antibodies at T1, T2, T3, and T4 in seronegative and natural seropositive subjects are shown both as aggregated results (**Figure 1B**) and after stratification in groups depending on different gender and age (**Figures 1C, D**). Overall, seronegative individuals had lower antibody titers than the seropositive group (**Figure 1B**) at all the observed time points (p<0.001).

#### 3.2.1 Serological evaluation at T1

Of the seronegative subjects (n=892) (i.e., without natural immunity against SARS-CoV-2), 881 (98.6%) had reactivity to the first vaccine dose. In contrast, 11 subjects showed an antibody titer below the cut-off value for reactivity, with a statistically significant difference between males (2.3%) and females (0.7%) (p=0.049). All non-responsive individuals were in older age groups (**Figure 1C**). All seropositive subjects demonstrated reactivity to the first vaccine dose (**Figures 1B, D**).

#### 3.2.2 Serological evaluation at T2

Among seronegative subjects, aggregated data (**Figure 1B**) showed an elicitation of immune response in 869 subjects out of 892 (97.4%), with a median value of 1642 U/ml (IQR: 913.5->2500). Two subjects showed an antibody titer below the cut-off for reactivity and were exclusively men (1 in the younger and 1 in the older group). Considering the disaggregated data among the responder subjects, the antibody titer appears to be influenced by age (**Figure 1C**), even if in a non-statistically significant manner. Significantly higher levels of antibody titer were observed in seropositive subjects compared to seronegative with a median of >2500 U/ml (upper level of instrument quantification) and 1642 U/ml, respectively (p<0.001, IQR 2500-2500 U/ml vs 913-2500 U/ml) (**Figure 1B**). No differences were observed at this time point among the different gender categories for seropositive subjects (**Figure 1D**).

#### 3.2.3 Serological evaluation at T3

Six months after vaccination (T3), seronegative subjects showed a reduction of antibody titer compared to the previous time-point (T2, p<0.001), with a median of 648 U/ml (IQR: 382-1108 U/ml) (**Figure 1B**). This value was lower compared to seropositive individuals who, at the same time point, showed mainly values above the instrument limit of quantification (p<0.001, median: ≥ 2500 U/ml) (**Figure 1B**). Among seronegative individuals, further reductions were observed in older subjects, especially males, with significant differences between this category and younger females (p=0.020) (**Figure 1C**). No decrease was observed in seropositive subjects, which showed median values above the 2500 U/mL instrument limit (**Figure 1B**). No age-based differences emerged among seronegative females (**Figure 1C**) or seropositive subjects (**Figure 1D**), for which also the gender did not exert any significant effect.

#### 3.2.4 Serological evaluation at T4

Ten months after vaccination, a further decrease was observed in the anti-RBD antibody titer of seronegative subjects compared to the previous time point as reported in **Figure 1B** (T3, p<0.001; median 428 U/ml, IQR 234-776). Again, specific age and gender characteristics were associated with a higher titer, with older males showing lower levels than younger females (p=0.048) (**Figure 1C**). A reduction was also observed at this time point in seropositive subjects, compared to T3 (p=0.046, median 2415, IQR 1363->2500 U/ml), yet they remained higher than seronegative individuals (p<0.001) (**Figure 1B**). So, among seronegative, the disaggregated evaluation of the antibody titers allowed the highlighting of differences between males and females and among different age groups, with a clear decreasing trend in antibody titers in the older age groups. Overall, the humoral responses were higher in females than in males, and a persistently higher antibody titer was related to pre-vaccination virus exposure with respect to seronegative subjects.

### 3.3 Influencing factors for the development of a robust humoral response

**Figure 2:**
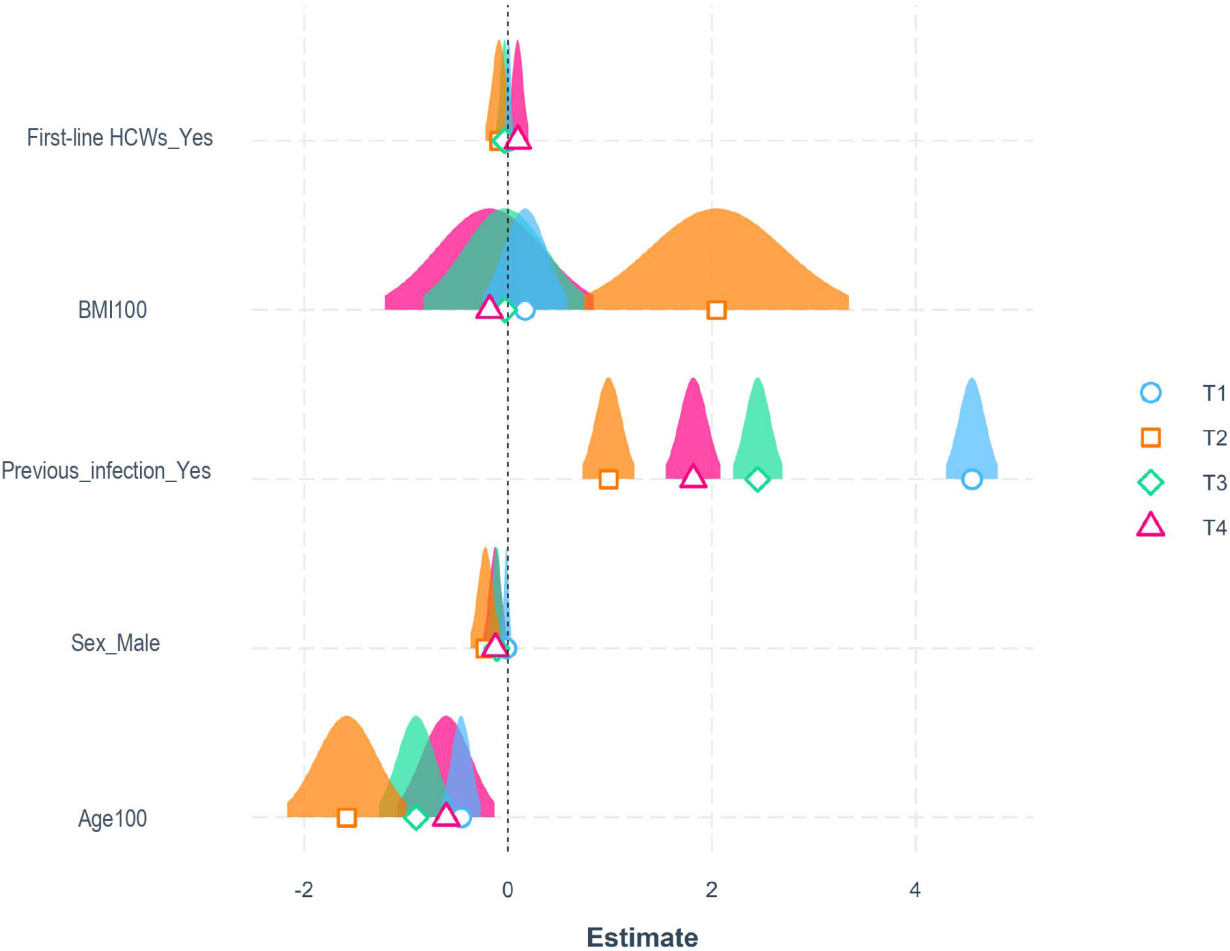
Influencing factors for the development of a robust humoral response. Representation of estimated coefficients associated with each variable in beta-regression models for antibody titers at T1 (21 days after the first dose) in blue, T2 (21 days after the second dose) in orange, T3 (6 months after the first dose) in green, and T4 (10 months after the first dose) in purple, for seronegative subjects. BMI and age were divided by 100 to enhance visualization (p values were identical to the original BMI and Age parameters).

Multiple beta-regression models for the assessment of the adjusted influence of different variables such as age, sex, Body Mass Index (BMI), comorbidities, previous infection, and professional category on antibody titer at different time points were designed. Due to the extremely higher antibody titer in natural seropositive compared to seronegative individuals, these categories have been separated for analysis. In seronegative subjects’ group, (**Figure 2**), younger age was associated with higher antibody titers at all time points analyzed (p<0.001), with an effect ranging from -2.1% (T2) to -1.0% (T4) in antibody titer per each 1% increase in age. Gender significantly influenced antibody titer at T2, T3, and T4, with males associated with lower values. BMI was associated with higher antibody titer at T2 only, with a 2.8% increase in antibody titer per 1% increase in BMI (**Figure 2**). First-line healthcare workers (i.e., physicians and nurses) showed T4 higher, yet not statistically significant, antibody titers than non-frontline workers at T4 (p=0.082). These analyses indicate that younger age and female gender are the main influencing factors for developing a robust and prolonged humoral response in vaccinated subjects. Conversely, the other parameters considered showed only trends for association with the magnitude of antibody response. With concern to seropositive subjects at baseline, no factors associated with humoral response emerged **Supplementary Table 2**.

### 3.4 Adverse reactions to the anti-SARS-CoV-2 vaccine

**Figure 3:**
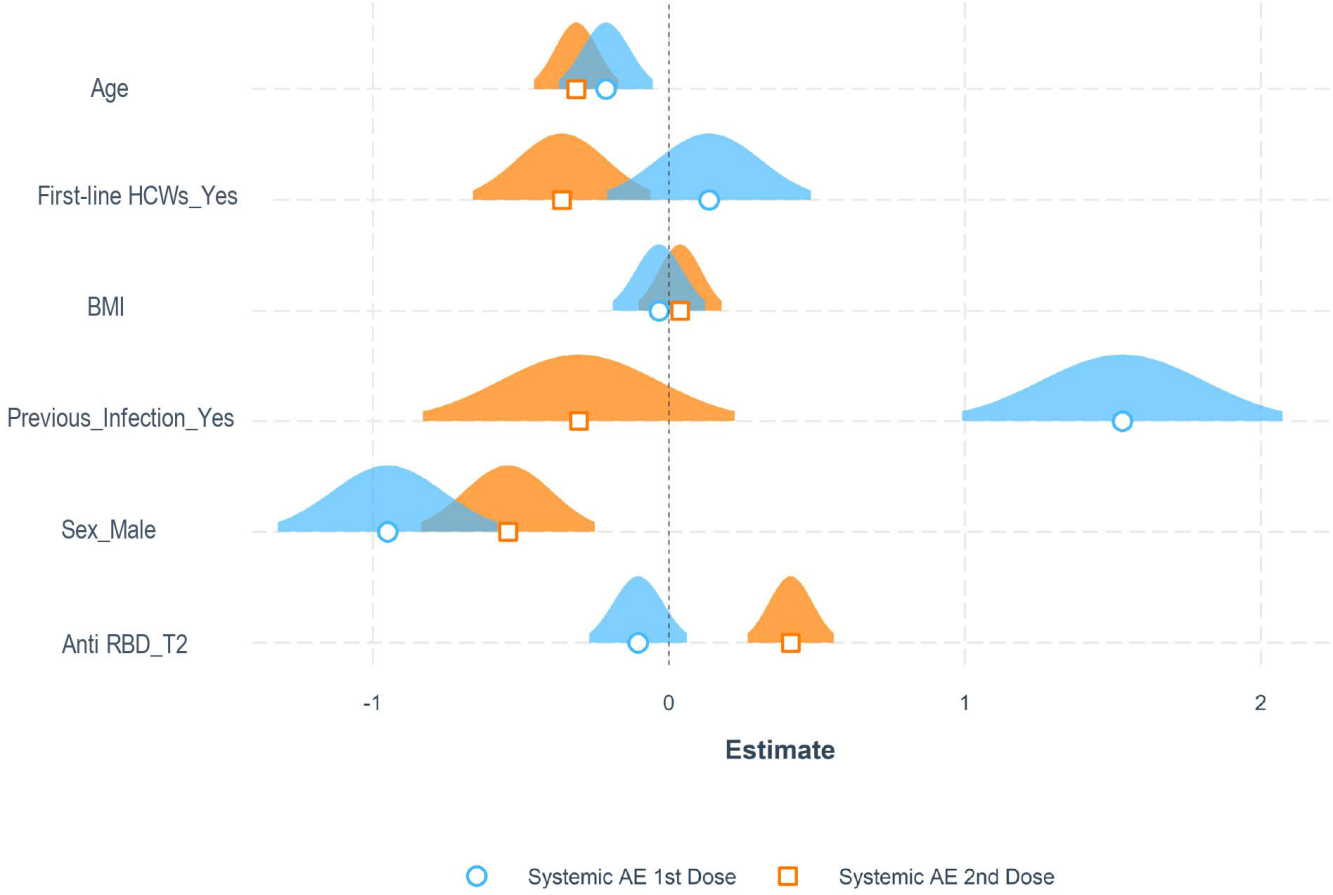
Adverse reactions to the anti-SARS-CoV-2 vaccine. Representation of estimated coefficients associated with each variable in multiple logistic regression models for the probability of experiencing systemic adverse events (AE) after the first and second dose in the entire cohort of patients.

Among the entire cohort, 684 (70.6%) and 735 (75.8%) subjects developed at least one adverse reaction following the first and second dose of the vaccine, respectively. Specifically, after the first dose, 635 (65.5%) participants reported localized reactions, 12 (1.2%) allergic reactions, 250 (25.8%) systemic reactions, and 211 (21.8%) other reactions **Supplementary Table 3**. On the other hand, after the second dose, 542 (55.9%) participants reported localized reactions, 17 (1.7%) allergic reactions, 522 (53.9%) systemic reactions, and 314 (32.4%) other reactions **Supplementary Table 4**. All reactions passed spontaneously after a few hours/days. Characteristics of patients who reported systemic reactions following vaccination are summarized in **Supplementary Table 5**. Multivariable logistic regression models were built to identify associated factors with systemic reactions following the first and the second doses of the vaccine. As selected by Akaike information criterion analysis, the final models included as covariates: previous SARS-CoV-2 infection, age, sex, professional category, BMI, and antibody titers at T2. Specifically, history of SARS-CoV-2 infection, younger age, and female sex were significantly associated with a higher incidence of systemic reactions after the first dose. Health-care-associated categories and higher antibody titers at T2 were associated with a higher probability of systemic reactions after the second dose (**Figure 3** and **Supplementary Table 6**).

### 3.5 SARS-CoV-2 specific T-cell response

**Figure 4:**
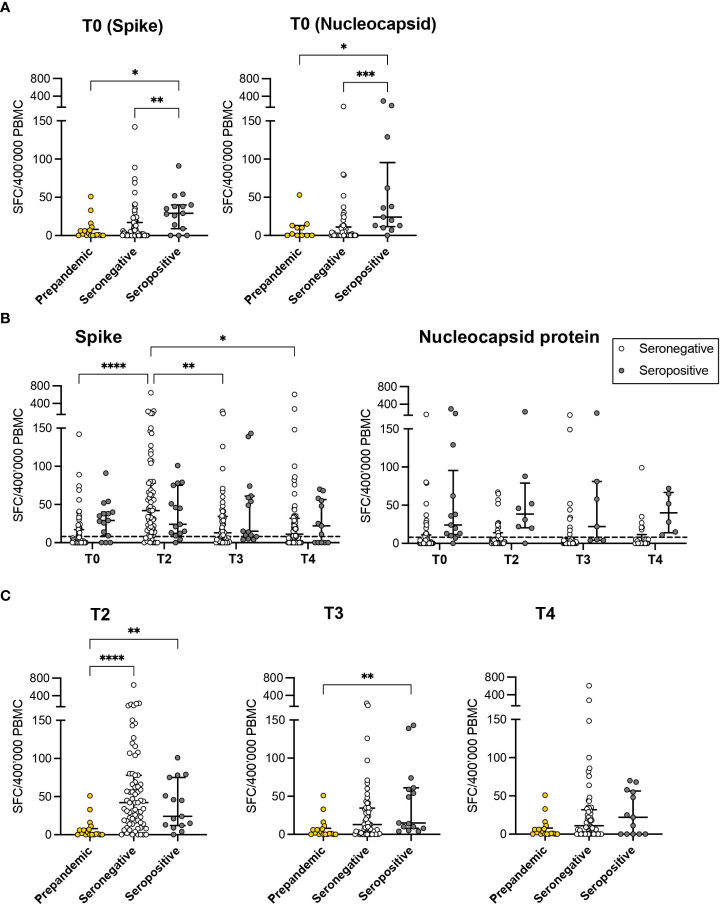
SARS-CoV-2 specific T-cell responses. **(A)** The magnitude of T-cell responses specific for both Spike and Nucleocapsid proteins in seropositive (grey) and seronegative (white) subjects was evaluated at T0 and compared to the results obtained from unexposed controls (prepandemic samples, yellow). **(B)** The kinetics of T-cell responses specific to the Spike and Nucleocapsid protein was evaluated at different time points after vaccination (T0, T2, T3, and T4) in seropositive and seronegative subjects. **(C)** Comparisons were reported between results obtained at different time points (T2, T3 and T4) from vaccinated subjects and from prepandemic samples. The results are expressed as specific Spot Forming Cells (SFC)\400’000 Peripheral Blood Mononuclear Cells (PBMC). *: p<0.05, **: p<0.01, ***: p<0.001, ****: p<0.0001. The dashed line represents the 75% percentile of the T-cell response in prepandemic samples.

We next longitudinally quantified the SARS-CoV-2 specific T-cell response after vaccination in all the evaluable subjects, as described in the Methods section. Natural seropositive subjects (n=18) demonstrated a superior T-cell response to the S protein (median 29 SFC/400’000 Peripheral Blood Mononuclear Cells [PBMC], IQR: 9-40) compared to pre-pandemic controls (n=17, median 1 SFC/400’000 PBMC, IQR: 0-8; p=0.0196) and seronegative individuals at baseline (T0) (n=89, median 2 SFC/400’000 PBMC, IQR: 0-17; p=0.008). No difference was observed between unexposed and seronegative subjects at T0 (p>0.999; **Figure 4A**). Accordingly, the T-cell response specific for the N protein was higher in natural seropositive subjects compared to those seronegative at T0 (p<0.001) and to unexposed controls (p<0.05; **Figure 4A**). After the vaccination, the frequency of S-specific T cells increased in seronegative subjects, reaching a peak at T2 (median 42, IQR: 16-78; p<0.0001). A slight but significant decrease was observed at T3 (median 13, IQR: 0-34; p<0.01) and T4 (median 11, IQR: 0-32; p<0.05). Seropositive subjects showed a similar magnitude of virus-specific T-cell responses at all time points. Interestingly, no significant difference emerged after T0 between seronegative and seropositive individuals (**Figure 4B**), indicating that the vaccination administered to naïve subjects elicits a SARS-CoV-2 specific T-cell response comparable to that observed in previously infected subjects who reported mild symptoms or were asymptomatic (26). As expected, while seronegative subjects displayed limited T-cell responses toward the Nucleocapsid protein at all timepoints, higher frequencies of N-specific T cells were observed in the cohort of seropositive subjects (**Figure 4B**). At each time-point, the level of S-specific T-cell response was compared between unexposed controls and samples collected from the vaccinated individuals (**Figure 4C**). The two vaccination doses elicited a SARS-CoV-2 specific T-cell response at T2 in both seronegative and seropositive individuals. At T3, only seropositive subjects showed significant enrichment of specific responses compared to unexposed subjects. Interestingly, at the last time point (T4), both groups showed a response contraction. Thus, the vaccination induced a robust SARS-CoV-2 specific T-cell response at early time points in subjects proving seronegative at baseline. However, a contraction was observed starting 6 months after the second dose in this group. On the other hand, the pre-existing response detected in natural seropositive subjects was further boosted and prolonged upon vaccination.

### 3.6 T-cell response and antibody titer correlation

**Figure 5:**
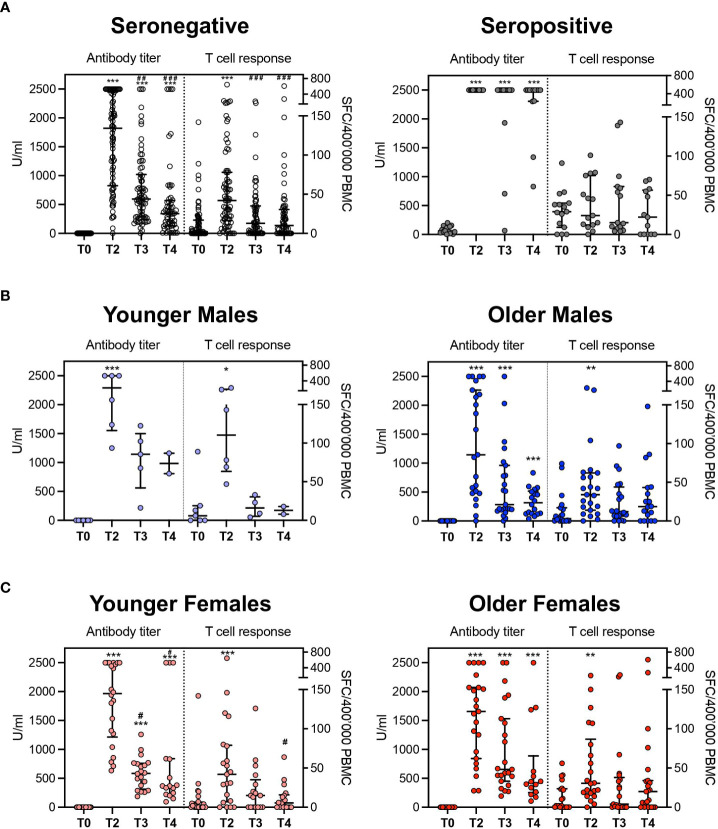
Comparison between antibody titer and T-cell response. **(A)** Aggregated results in seronegative and seropositive subjects. **(B)** disaggregated data by age groups (seronegative males). **(C)** disaggregated data by age groups (seronegative females). The antibody titer is expressed in U/ml (unit/milliliter) and T cellular response is expressed as specific Spot Forming Cells (SFC)\400’000 Peripheral Blood Mononuclear Cells (PBMC). *p<0.05, **p<0.01 and ***p<0.001 vs T0; #p<0.05, ##p<0.01 and ###p<0.001 vs T2.

As the antibody titers and the frequency of specific T cells were assessed in parallel on the same samples, we next wondered whether the two main branches of the adaptive response, the humoral and cell-mediated ones, were stimulated with similar kinetics. In seronegative subjects, no significant correlation was observed at any time point between antibody titers and frequencies of IFN-γ producing specific T cells using Spearman’s method. Nevertheless, considering that antibody titer and frequency of specific T-cell response showed a beta distribution, after proper transformation of values, we observed that in a beta-regression model, the level of anti-S antibody was significantly associated with T cells response specific for the S-protein at T4. However, this was not confirmed for previous time points. No association was observed for what concerns seropositive subjects (**Supplementary Image 1**). Despite this, antibody titers and specific T-cell responses expanded upon vaccination, reaching a peak at T2 in seronegative subjects and contracting at later timepoints (p<0.001, **Figure 5A**). Differently, the humoral response was remarkably expanded at all timepoints compared to the baseline. The T-cell responses were stably high across the different timepoints in the cohort of seropositive subjects (**Figure 5A**). Using the same models, no influence of age, gender, and other variables (BMI, comorbidities, previous infections, occupation) emerged concerning the T-cell response. Indeed, among seronegative subgroup, the T-cell response varied significantly between T0 and T2 in all age and gender categories and then contracted (**Figures 5B, C**). Similarly, in the ELISpot population, antibody titers peaked at T2. Interestingly, while males experienced a rapid decrease of humoral responses afterward, females showed significant enrichment of antibody titers till the last time-point (T4) compared to the baseline evaluation, confirming a more prolonged reactivity to viral antigens after vaccination as observed in the entire cohort.

## 4 Discussion

SARS-CoV-2 mRNA vaccines elicit both virus-specific humoral and T-cell responses (14). However, host protection is ensured by a complex interaction of several influencing factors, such as natural immunity, gender, and age. Modifying one of these variables can alter the degree of immunization achieved with vaccination. It should consequently change the chosen assay to evaluate individual responses, placing specific features and needs in the foreground. Serological assays, currently available to assess anti-SARS-CoV-2 antibodies, help test large cohorts due to cost-effectiveness, and ability to process a large amount of data in a brief turnaround time. Still, their applicability is limited as it has not had the potential to reflect neutralizing activity *in vitro* and is not applicable alone to evaluate the anti-SARS-CoV-2 immunization after vaccination (8, 9).

Moreover, assessing seroprevalence in a heterogenous population may be difficult due to a documented decline that occurs over time starting from 3 months after vaccination (27). Our findings demonstrated a reduced humoral response 6 months after vaccination, lessening at 10 months, in line with literature data (28, 29). Additionally, to assess a solid correlate of protection, the involvement of other immunological mechanisms, such as long-lived memory B cells and T cells, are to be considered (10). We potentiate our findings by testing T-cell response to virus-specific peptides to overcome serology testing limitations. Natural seropositive subjects maintained higher values than those observed in seronegative individuals even in the latest time points, denoting that SARS-CoV-2 infection entails an enduring humoral response. This suggested that antibody testing remains a valuable companion diagnostic tool, especially for individuals who have already been exposed to the virus and who benefit from a longer-lasting specific antibody titer persistence.

Moreover, these data confirm that a memory B cell pool is elicited after infection and is reinvigorated by vaccination (14, 29). On the contrary, age and gender slightly impact this category’s magnitude of humoral response, showing that the history of infection has a more substantial effect than other investigated variables. In seronegative individuals, a higher response was found in younger females compared to older males at 6 and 10 months after administration. As observed for other vaccinations, females develop more significant humoral responses than males due to the opposite effect on B cells exerted by estrogens and progesterone/androgens. While estrogens promote antibody production, mediated by Th2-associated cytokines IL-4 and IL-5 driving B cell proliferation and differentiation to plasma cells, progesterone, and androgens display inhibitory effects on B-cell response (30). So, since the male sex is associated with severe COVID-19 and death, accurate detection of immune coverage appears to be pivotal in these individuals (31) to plan an effective vaccination schedule. Furthermore, younger age is associated with higher antibody titers at all the analyzed time points, confirming that immunosenescence affects the B compartment both in females and males (18) and determines a decline in humoral response with aging. Alterations in B-cell repertoire in the elderly may explain this waning in humoral immune function, such as defects in B lymphopoiesis, cell development, and homeostasis (32). Consequently, serological tests may not accurately capture the immunization status of older subgroups of the vaccinated population, limiting their applicability to real-world scenarios. Moreover, the female sex, younger age, and previously infected subjects are more associated with adverse reactions to vaccination. Therefore, in planning optimized subsequent doses administrations, these categories may need closer monitoring of side effects, especially systemic ones.

T-cell response assays could provide complementary information to those inferred by serological tests for a full-wide view of the immunity elicited by vaccination. The current automation and standardization of T-cell assays (33) have paved the path for their applicability to broader populations. Nevertheless, the ELISpot assay needs skilled laboratory personnel and complex procedures. Moreover, SARS-CoV-2-specific T-cell evaluations may be affected by cross-reactivity toward other coronaviruses (31, 34). To overcome this potential bias, we tested PBMCs collected from healthy donors before 2019. Our data showed that seropositive subjects display a superior T-cell response at baseline than seronegative subjects and unexposed controls, attesting that the ELISpot assay is highly sensitive, even when the rate of response is low. Yet, the BNT162b2 vaccination induced differential T-cell response kinetics in seronegative and seropositive subjects. Indeed, the effect of a previous SARS-CoV-2 infection is noteworthy since seropositive individuals show a longer-lasting persistence of specific T-cell response when compared to unexposed subjects (T3), indicating that the T-cell memory pool elicited by the infection (35) is reinvigorated by vaccine administration. Considering uninfected individuals at baseline, specific T-cell responses peaked after the second dose but then contracted, starting 6 months after vaccination. This evidence is consistent with a decline in T-cell mediated response over time observed by others (34, 36, 37). By analogy with humoral response data, we analyzed potential influencing factors amongst demographic and anthropometric characteristics collected on participants by the survey. Disaggregated data analysis revealed a limited impact of gender on specific T-cell responses. Indeed, similar values were observed between males and females at all timepoints. Under our findings, other authors failed to observe a gender effect (35, 37, 38), except for Costa et al. (34), who showed a reduced cellular response in males compared to females. This is possibly due to the broader population considered, which better displays the inhibitory role exerted by androgens on TH1 cells (39). The role of age is more controversial. Costa et al. (34) reported that the level of response was directly correlated with increasing age; however, other studies performed on healthcare workers found a trend for a contracted response in the elderly, with differences decreasing after the second dose (40). In our cohort, age plays no substantial role in determining the magnitude of the cellular response, as we registered no difference between younger and older groups. This is probably due to a more homogeneous age distribution, not including the elderly.

As the detection of humoral and T-cell responses may differ based on subjects’ characteristics, we compared the kinetics of both humoral and T-cell responses over time. This correlation appears controversial in literature (13, 35). Some studies have evaluated T-cell and humoral responses after two doses of the BNT162b2 vaccine despite being mainly focused on recently vaccinated individuals or shorter follow up (13, 34, 35, 37, 38, 41–44), different age categories (40), and smaller populations (13, 35, 37, 38, 42, 43, 45, 46). Of note, in our study, the two branches did not correlate at any time point considered. We infer that serological tests and T-cell response assays report complementary information which could accurately depict the individual immunization status, compensating for each other’s limitations. Thus, when serological tests fail to identify immune coverage, T-cell assays may find a diagnostic application, e.g., in case of treatments based on the administration of anti-CD20 antibodies (47) or other conditions inducing B-cell aplasia (31) or in case of individuals not displaying a detectable antibody titer or displaying a reduced antibody response (old males). Furthermore, in frail patients or high-risk working categories who should be carefully monitored for protection, both tests appear essential to assess the immunization status accurately, in line with Seraceni et al. (13).

Therefore, the survey could represent a valuable tool to highlight potential influencing factors on the longevity and magnitude of vaccinees’ adaptive responses. Indeed, it could suggest careful monitoring of high-risk subjects and timing for boostering for those with factors causing a significant waning in immunity. Other studies identified possible immune variability predictors yet considered their relative impact in shorter time framings (34, 41, 48), not evaluating gender differences (48) or focusing mainly on their impact on the humoral response (49, 50) in different population settings (50). Further studies will be needed to validate the findings in a broader population.

In conclusion, vaccine administrations should not be considered one-size-fits-all (51) but should be targeted to individual immunization status. The antibody titer detection alone is informative, as it benefits from a fast and widely applicable method. Still, it is limited as it lacks a standardized threshold for protection from reinfection. With the increasing number of automized and CE-approved assays for the cellular response, the T-cell response may play a complementary role in selected settings, exploring the other arm of adaptive immunity. Age and gender are important determinants that tip the balance of equality of immunization between large cohorts and need to be investigated deeper. This evidence opens future insights into rescheduling immunization status-dependent administrations by considering individuals’ differences in response and foreshadows the idea of an immunization passport based on age, gender, antibody titer, and cellular response.

## Data availability statement

The original contributions presented in the study are included in the article/**Supplementary Material**. Further inquiries can be directed to the corresponding authors.

## Ethics statement

The studies involving human participants were reviewed and approved by Ospedale San Raffaele- Ethical Committee. The study was conducted according to the guidelines of the Declaration of Helsinki. The patients/participants provided their written informed consent to participate in this study.

## Author contributions

GB, RT, PRQ, CB, and FC designed the study. ES, MN, DF, CDR, VV, VB, and CS conducted laboratory experiments, analyzed and interpreted data. MV, MN, and RDL performed statistical analysis. ES, MN, MV, and RDL wrote the paper. ML and GB participated to data discussion and interpretation. PRQ, CB, and RT supervised the study and wrote the paper. All authors contributed to the article and approved the submitted version.
