# Supplementary material for: A longitudinal analysis of humoral, T cellular response and influencing factors in a cohort of healthcare workers: Implications for personalized SARS-CoV-2 vaccination strategies
Source: Front Immunol. 2023 Mar 14;14:1130802. doi: 10.3389/fimmu.2023.1130802 (PMC10043299; doi:10.3389/fimmu.2023.1130802)

Supplementary Figure 1*. Correlation plots between anti-RBD antibody titer (U/ml) and SFC/400'000 PBMC at different timepoints in seropositive and seronegative subjects.*

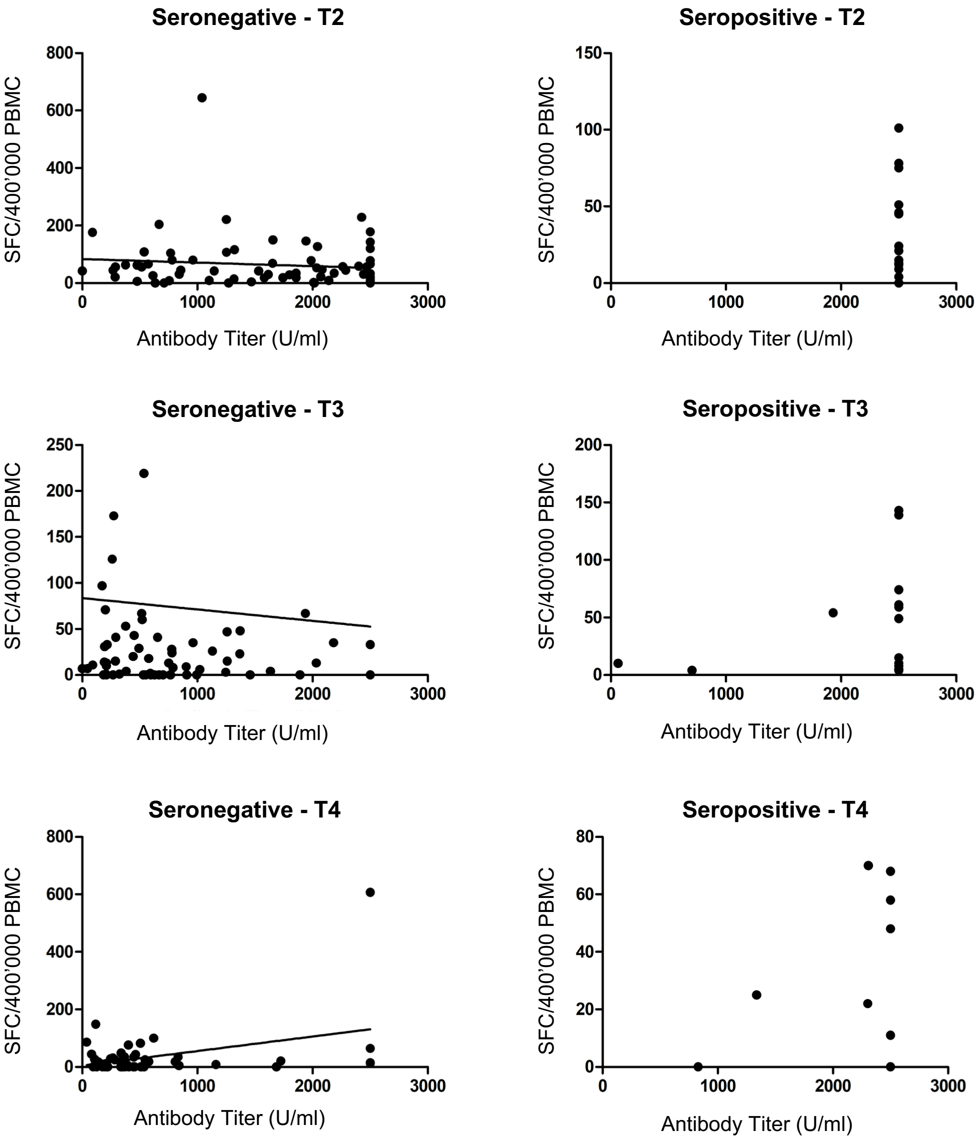

Supplement: Supplementary file 1 [file Image_1.pdf]
