# Supplementary material for: A longitudinal analysis of humoral, T cellular response and influencing factors in a cohort of healthcare workers: Implications for personalized SARS-CoV-2 vaccination strategies
Source: Front Immunol. 2023 Mar 14;14:1130802. doi: 10.3389/fimmu.2023.1130802 (PMC10043299; doi:10.3389/fimmu.2023.1130802)
Supplement: Supplementary file 2 [file Table_1.docx]

***Supplementary Table 1****. General features of the population analyzed for humoral responses*

|  | Overall (n=969) |
| --- | --- |
| Age, years | 51 (41-58) |
| BMI | 23.4 (21.1-26.2) |
| Previous SARS-CoV-2 infection | 77 (7.9) |
| Female sex | 634 (65.4) |
| Etnicity   - Asian - White - Hispanic or Latino | 5 (0.5)  947 (97.7)  11 (1.1) |
| Smoking | 108 (11.1) |
| Comorbidities   - Type I DM - Type II DM - Active neoplasia - CVD - Immunodepression or history of organ transplant - Autoimmune disease - Neurological disease - History of allergy   Coagulopathy | 4 (0.4)  11 (1.1)  17 (1.8)  195 (20.1)  5 (0.5)  77 (7.9)  11 (1.1)  49 (5.1)  7 (0.7) |

Categorical variables were expressed as absolute count (%), while continuous variables as median (IQR). Abbreviations. BMI, body mass index. DM, diabetes mellitus. CVD, cardiovascular disease.
