# Supplementary material for: A longitudinal analysis of humoral, T cellular response and influencing factors in a cohort of healthcare workers: Implications for personalized SARS-CoV-2 vaccination strategies
Source: Front Immunol. 2023 Mar 14;14:1130802. doi: 10.3389/fimmu.2023.1130802 (PMC10043299; doi:10.3389/fimmu.2023.1130802)
Supplement: Supplementary file 3 [file Table_2.docx]

***Supplementary Table 2*.** *Results of beta regression models for anti-RBD antibody titer at different timepoints*

| **T1** | | | | | |
| --- | --- | --- | --- | --- | --- |
|  | Estimate | Std. Error | z value | Pr(>\|z\|) |  |
| (Intercept) | -0.9 | 0.1 | -12838.0 | < 2e-16 | *** |
| Age100 | -0.5 | 0.1 | -4766.0 | 0.000 | *** |
| Sex_Male | 0.0 | 0.0 | -0.8 | 0.445 |  |
| Previous_infection_Yes | 4.6 | 0.1 | 35.2 | < 2e-16 | *** |
| BMI100 | 0.2 | 0.2 | 0.8 | 0.429 |  |
| First-line_HCWs_Yes | 0.0 | 0.0 | 0.2 | 0.810 |  |
| Type of estimator: ML (maximum likelihood) | | | | | |
| Log-likelihood: 2233 on 7 Df | | | | | |
| Pseudo R-squared: 0.8717 | | | | | |
| Number of iterations: 28 (BFGS) + 2 (Fisher scoring) | | | | | |
| **T2** | | | | | |
|  | Estimate | Std. Error | z value | Pr(>\|z\|) |  |
| (Intercept) | 1.5 | 0.2 | 7030.0 | 0.000 | *** |
| Age100 | -1.6 | 0.3 | -5294.0 | 0.000 | *** |
| Sex_Male | -0.2 | 0.1 | -3021.0 | 0.003 | ** |
| Previous_infection_Yes | 1.0 | 0.1 | 7606.0 | 0.000 | *** |
| BMI100 | 2.0 | 0.7 | 3087.0 | 0.002 | ** |
| First-line_HCWs_Yes | -0.1 | 0.1 | -1292.0 | 0.196 |  |
| Type of estimator: ML (maximum likelihood) | | | | | |
| Log-likelihood: 1106 on 7 Df | | | | | |
| Pseudo R-squared: 0.1754 | | | | | |
| Number of iterations: 27 (BFGS) + 3 (Fisher scoring) | | | | | |
| **T3** | | | | | |
|  | Estimate | Std. Error | z value | Pr(>\|z\|) |  |
| (Intercept) | 0.4 | 0.1 | 3493.0 | 0.000 | *** |
| age100 | -0.9 | 0.2 | -4870.0 | 0.000 | *** |
| Sex_Male | -0.1 | 0.0 | -2502.0 | 0.012 | * |
| Previous_infection_Yes | 2.5 | 0.1 | 19.8 | < 2e-16 | *** |
| BMI100 | 0.0 | 0.4 | -0.1 | 0.938 |  |
| First-line_HCWs_Yes | 0.0 | 0.0 | -0.8 | 0.416 |  |
| Type of estimator: ML (maximum likelihood) | | | | | |
| Log-likelihood: 358 on 7 Df | | | | | |
| Pseudo R-squared: 0.4776 | | | | | |
| Number of iterations: 26 (BFGS) + 1 (Fisher scoring) | | | | | |
| **T4** | | | | | |
|  | Estimate | Std. Error | z value | Pr(>\|z\|) |  |
| (Intercept) | 0.3 | 0.2 | 1681.0 | 0.093 | . |
| age100 | -0.6 | 0.2 | -2490.0 | 0.013 | * |
| Sex_Male | -0.1 | 0.1 | -2178.0 | 0.029 | * |
| Previous_infection_Yes | 1.8 | 0.1 | 13.3 | <2e-16 | *** |
| BMI100 | -0.2 | 0.5 | -0.4 | 0.725 |  |
| First-line_HCWs_Yes | 0.1 | 0.1 | 1738.0 | 0.082 | . |
| Type of estimator: ML (maximum likelihood) | | | | | |
| Log-likelihood: 263.2 on 7 Df | | | | | |
| Pseudo R-squared: 0.2747 | | | | | |
| Number of iterations: 20 (BFGS) + 3 (Fisher scoring) | | | | | |
