# Supplementary material for: A longitudinal analysis of humoral, T cellular response and influencing factors in a cohort of healthcare workers: Implications for personalized SARS-CoV-2 vaccination strategies
Source: Front Immunol. 2023 Mar 14;14:1130802. doi: 10.3389/fimmu.2023.1130802 (PMC10043299; doi:10.3389/fimmu.2023.1130802)
Supplement: Supplementary file 4 [file Table_3.docx]

***Supplementary Table 3.*** *Reactions to the first dose of anti-SARS-CoV-2 vaccine.*

| Reactions to the first dose of the anti-SARS-CoV-2 vaccine | Overall (n=969) | No history of SARS-CoV-2 infection (n=892) | History of SARS-CoV-2 infection (n=77) | P value |
| --- | --- | --- | --- | --- |
| *Localized reactions* |  |  |  |  |
| Pain at the injection site | 616 (63.6) | 562 (63) | 54 (70.1) | 0.49 |
| Swelling at the injection site | 119 (12.3) | 107 (12) | 12 (15.6) | 0.48 |
| *Systemic reactions* |  |  |  |  |
| Fever | 28 (2.9) | 16 (1.8) | 12 (15.6) | <0.001 |
| Tiredness/malaise | 206 (21.3) | 169 (18.9) | 37 (48.1) | <0.001 |
| Chills | 55 (5.7) | 43 (4.8) | 12 (15.6) | <0.001 |
| Myalgias | 98 (10.1) | 76 (8.5) | 22 (28.6) | <0.01 |
| Arthralgias | 76 (7.8) | 62 (7) | 14 (18.2) | 0.0022 |
| *Allergic reactions* |  |  |  |  |
| Widespread itching | 5 (0.5) | 5 (0.5) | 0 (0) | 0.80 |
| Cutaneous rash | 9 (0.9) | 9 (1) | 0 (0) | 0.78 |
| Asthma | 0 | 0 | 0 | - |
| Throat tightness | 3 (0.3) | 2 (0.2) | 1 (1.3) | 0.58 |
| Anaphylaxis | 0 | 0 | 0 | - |
| *Other reactions* |  |  |  |  |
| Vomiting/nausea | 20 (2.1) | 14 (1.6) | 6 (7.8) | 0.001 |
| Diarrhoea | 9 (0.9) | 9 (1) | 0 (0) | 0.78 |
| Swollen lymph nodes | 17 (1.8) | 16 (1.8) | 1 (1.3) | >0.99 |
| Headache | 109 (11.2) | 96 (10.8) | 13 (16.9) | 0.16 |
| Dizziness/confusion | 27 (2.8) | 21 (2.4) | 6 (7.8) | 0.020 |
| Sleep quality alteration | 18 (1.9) | 14 (1.6) | 4 (5.2) | 0.071 |
| Memory loss | 1 (0.1) | 0 (0) | 1 (1.3) | 0.12 |
| Anxiety | 4 (0.4) | 3 (0.3) | 1 (1.3) | 0.74 |
| Psychological stress | 5 (0.5) | 4 (0.4) | 1 (1.3) | 0.87 |
| Feeling of gratitude/relief/joy | 50 (5.2) | 48 (5.4) | 2 (2.6) | 0.42 |
| Attention deficit | 9 (0.9) | 9 (1) | 0 (0) | 0.78 |
| Palpitations | 14 (1.4) | 11 (1.2) | 3 (3.9) | 0.17 |
| Chest pain | 2 (0.2) | 1 (0.1) | 1 (1.3) | 0.37 |
| Appetite loss | 5 (0.5) | 4 (0.4) | 1 (1.3) | 0.87 |
| Increased thirst | 5 (0.5) | 4 (0.4) | 1 (1.3) | 0.87 |
| Heat/cold intolerance | 3 (0.3) | 1 (0.1) | 2 (2.6) | 0.0073 |
| Menstrual cycle alterations | 5 (0.5) | 5 (0.6) | 0 (0) | >0.99 |
| Difficulty in performing daily life activities | 14 (1.4) | 10 (1.1) | 4 (5.2) | 0.018 |
| Categorical variables were expressed as absolute count (%). | | | | |
