# Supplementary material for: A longitudinal analysis of humoral, T cellular response and influencing factors in a cohort of healthcare workers: Implications for personalized SARS-CoV-2 vaccination strategies
Source: Front Immunol. 2023 Mar 14;14:1130802. doi: 10.3389/fimmu.2023.1130802 (PMC10043299; doi:10.3389/fimmu.2023.1130802)
Supplement: Supplementary file 5 [file Table_4.docx]

***Supplementary Table 4.*** *Reactions to the second dose of anti-SARS-CoV-2 vaccine.*

| Reactions to the second dose of anti-SARS-CoV-2 vaccine | Overall (n=969) | No history of SARS-CoV-2 infection (n=892) | History of SARS-CoV-2 infection (n=77) | P value |
| --- | --- | --- | --- | --- |
| *Localized reactions* |  |  |  |  |
| Pain at the injection site | 526 (54.3) | 485 (54.4) | 41 (53.2) | 0.92 |
| Swelling at the injection site | 106 (10.9) | 95 (10.7) | 11 (14.3) | 0.45 |
| *Systemic reactions* |  |  |  |  |
| Fever | 190 (19.6) | 169 (18.9) | 21 (27.3) | 0.11 |
| Tiredness/malaise | 430 (44.4) | 390 (43.7) | 40 (51.9) | 0.23 |
| Chills | 173 (17.9) | 158 (17.7) | 15 (19.5) | 0.85 |
| Myalgias | 256 (26.4) | 229 (25.7) | 27 (35.1) | 0.11 |
| Arthralgias | 195 (20.1) | 173 (19.4) | 22 (28.6) | 0.082 |
| *Allergic reactions* |  |  |  |  |
| Widespread itching | 12 (1.2) | 11 (1.2) | 1 (1.3) | >0.99 |
| Cutaneous rash | 9 (0.9) | 9 (1) | 0 (0) | 0.78 |
| Asthma | 0 | 0 | 0 | - |
| Throat tightness | 4 (0.4) | 2 (0.2) | 2 (2.6) | 0.029 |
| Anaphylaxis | 1 (0.1) | 1 (0.1) | 0 (0) | >0.99 |
| *Other reactions* |  |  |  |  |
| Vomiting/nausea | 38 (3.9) | 34 (3.8) | 4 (5.2) | 0.78 |
| Diarrhoea | 13 (1.3) | 13 (1.5) | 0 (0) | 0.58 |
| Swollen lymph nodes | 44 (4.5) | 42 (4.7) | 2 (2.6) | 0.56 |
| Headache | 187 (19.3) | 169 (18.9) | 18 (23.4) | 0.64 |
| Dizziness/confusion | 51 (5.3) | 48 (5.4) | 3 (3.9) | 0.75 |
| Sleep quality alteration | 26 (2.7) | 21 (2.4) | 5 (6.5) | 0.077 |
| Memory loss | 11 (1.1) | 7 (0.8) | 4 (5.2) | 0.0034 |
| Anxiety | 7 (0.7) | 5 (0.6) | 2 (2.6) | 0.19 |
| Psychological stress | 4 (0.4) | 4 (0.4) | 0 (0) | >0.99 |
| Feeling of gratitude/relief/joy | 47 (4.9) | 45 (5) | 2 (2.6) | 0.48 |
| Attention deficit | 18 (1.9) | 16 (1.8) | 2 (2.6) | 0.96 |
| Palpitations | 11 (1.1) | 7 (0.8) | 4 (5.2) | 0.0034 |
| Chest pain | 8 (0.8) | 6 (0.7) | 2 (2.6) | 0.25 |
| Appetite loss | 7 (0.7) | 6 (0.7) | 1 (1.3) | >0.99 |
| Increased thirst | 7 (0.7) | 5 (0.6) | 2 (2.6) | 0.19 |
| Heat/cold intolerance | 9 (0.9) | 6 (0.7) | 3 (3.9) | 0.028 |
| Menstrual cycle alterations | 7 (0.7) | 7 (0.8) | 0 (0) | 0.93 |
| Difficulty in performing daily life activities | 32 (3.3) | 28 (3.1) | 4 (5.2) | 0.53 |
| Categorical variables were expressed as absolute count (%). | | | | |
