# Supplementary material for: A longitudinal analysis of humoral, T cellular response and influencing factors in a cohort of healthcare workers: Implications for personalized SARS-CoV-2 vaccination strategies
Source: Front Immunol. 2023 Mar 14;14:1130802. doi: 10.3389/fimmu.2023.1130802 (PMC10043299; doi:10.3389/fimmu.2023.1130802)
Supplement: Supplementary file 6 [file Table_5.docx]

***Supplementary Table 5****. Characteristics of patients who experienced systemic reactions following*

*the first and second dose of the vaccine.*

|  | Systemic reactions following the first dose (n=250) | Systemic reactions following the second dose (n=522) |
| --- | --- | --- |
| Age, years | 48 (37.2-55) | 49 (38-56) |
| Female sex | 198 (79.2) | 372 (71.3) |
| BMI, Kg/m^2^ | 22.7 (20.4-25.3) | 23.2 (20.9-26) |
| Previous SARS-CoV-2 infection | 40 (16) | 44 (8.4) |
| *Ethnicity* |  |  |
| Asian | 1 (0.4) | 3 (0.6) |
| Hispanic or Latino | 7 (2.8) | 3 (0.6) |
| White | 242 (96.8) | 516 (98.8) |
| Smoking | 31 (12.4) | 59 (11.3) |
| *Comorbidities* |  |  |
| Type I DM | 1 (0.4) | 0 |
| Type II DM | 2 (0.8) | 8 (1.5) |
| Active neoplasia | 5 (2) | 9 (1.7) |
| CVD | 45 (18) | 99 (19) |
| Immunodepression or history of organ transplant | 0 | 1 (0.2) |
| Autoimmune disease | 18 (7.2) | 44 (8.4) |
| Neurological disease | 2 (0.8) | 8 (1.5) |
| History of allergy | 17 (6.8) | 30 (5.7) |
| Coagulopathy | 3 (1.2) | 4 (0.8) |
| *Signs or symptoms following vaccination* |  |  |
| Fever | 26 (10.4) | 190 (36.4) |
| Tiredness/malaise | 204 (81.6) | 428 (82) |
| Chills | 53 (21.2) | 172 (33) |
| Myalgias | 97 (38.8) | 251 (48.1) |
| Arthralgias | 75 (30) | 192 (36.8) |
| Categorical variables were expressed as absolute count (%), while continuous variables as median (IQR). Abbreviations. BMI, body mass index. DM, diabetes mellitus. CVD, cardiovascular disease. | | |
