# Supplementary material for: A longitudinal analysis of humoral, T cellular response and influencing factors in a cohort of healthcare workers: Implications for personalized SARS-CoV-2 vaccination strategies
Source: Front Immunol. 2023 Mar 14;14:1130802. doi: 10.3389/fimmu.2023.1130802 (PMC10043299; doi:10.3389/fimmu.2023.1130802)
Supplement: Supplementary file 7 [file Table_6.docx]

***Supplementary Table 6.*** *Results of logistic regression models for systemic AE after 1^st^ and 2^nd^ doses.*

| Systemic AE after 1st dose | | | | | |
| --- | --- | --- | --- | --- | --- |
|  | Estimate | Std. Error | z value | Pr(>\|z\|) |  |
| (Intercept) | 0.23 | 0.52 | 0.44 | 0.663 |  |
| antiRBD_T2 | 0.00 | 0.00 | -1243.00 | 0.214 |  |
| Sex_Male | -0.95 | 0.19 | -5027.00 | 0.000 | *** |
| Previous_infection_Yes | 1.53 | 0.28 | 5545.00 | 0.000 | *** |
| BMI | -0.01 | 0.02 | -0.42 | 0.671 |  |
| Firstline_HCWs_Yes | 0.13 | 0.18 | 0.77 | 0.441 |  |
| Age | -0.02 | 0.01 | -2642.00 | 0.008 | ** |
|  |  |  |  |  |  |
| Null deviance: 1064.48 on 933 degrees of freedom; Residual deviance: 996.16 on 927 degrees of freedom; AIC: 1010.2 | | | | | |
| Systemic AE after 2nd dose | | | | | |
|  | Estimate | Std. Error | z value | Pr(>\|z\|) |  |
| (Intercept) | 9.17E+02 | 4.69E+02 | 1.96 | 0.050 | . |
| antiRBD_T2 | 5.18E-01 | 9.32E-02 | 5.56 | 0.000 | *** |
| Sex_Male | -5.44E+02 | 1.50E+02 | -3.63 | 0.000 | *** |
| Previous_infection_Yes | -3.04E+02 | 2.68E+02 | -1.13 | 0.257 |  |
| BMI | 6.99E+00 | 1.35E+01 | 0.52 | 0.606 |  |
| First line_HCWs_Yes | -3.63E+02 | 1.53E+02 | -2.37 | 0.018 | * |
| Age | -2.69E+01 | 6.24E+00 | -4.31 | 0.000 | *** |
| Null deviance: 1289.2 on 933 degrees of freedom; Residual deviance: 1201.3 on 927 degrees of freedom; AIC: 1215.3 | | | | | |
